# Establishment and mitotic stability of an extra-chromosomal mammalian replicon

**DOI:** 10.1186/1471-2121-8-33

**Published:** 2007-08-06

**Authors:** Isa M Stehle, Jan Postberg, Sina Rupprecht, Thomas Cremer, Dean A Jackson, Hans J Lipps

**Affiliations:** 1 Institute of Cell Biology, University Witten/Herdecke, Stockumer Str. 10, 58453 Witten, Germany; 2 Department of Biology II, Anthropology and Human Genetics, Ludwig Maximilians University Munich, Großhaderner Straße 2, 82152 Martinsried, Germany; 3 Faculty of Life Sciences, University of Manchester, Manchester, Sackville Street, Manchester, M60 1QD, UK

## Abstract

**Background:**

Basic functions of the eukaryotic nucleus, like transcription and replication, are regulated in a hierarchic fashion. It is assumed that epigenetic factors influence the efficiency and precision of these processes. In order to uncouple local and long-range epigenetic features we used an extra-chromosomal replicon to study the requirements for replication and segregation and compared its behavior to that of its integrated counterpart.

**Results:**

The autonomous replicon replicates in all eukaryotic cells and is stably maintained in the absence of selection but, as other extra-chromosomal replicons, its establishment is very inefficient. We now show that following establishment the vector is stably associated with nuclear compartments involved in gene expression and chromosomal domains that replicate at the onset of S-phase. While the vector stays autonomous, its association with these compartments ensures the efficiency of replication and mitotic segregation in proliferating cells.

**Conclusion:**

Using this novel minimal model system we demonstrate that relevant functions of the eukaryotic nucleus are strongly influenced by higher nuclear architecture. Furthermore our findings have relevance for the rational design of episomal vectors to be used for genetic modification of cells: in order to improve such constructs with respect to efficiency elements have to be identified which ensure that such constructs reach regions of the nucleus favorable for replication and transcription.

## Background

Basic functions of the eukaryotic nucleus, like transcription and replication, are regulated in a hierarchic fashion and it is assumed that epigenetic factors influence the precision and efficiency of these processes [1]. Epigenetic regulators operate at many levels such as post-translational modifications of histone proteins that define a histone code [2], the 3D architecture and dynamic properties of chromatin domains and chromosome territories [3], and the interaction of chromatin with nuclear compartments that form dedicated sites of nuclear function [1,4]. The regulatory potential that results from the combination of these features is clearly complex, and as a result it is extremely challenging to define how each feature might influence chromatin function. One approach to address this problem is to locally uncouple the different epigenetic features.

**Figure 1:**
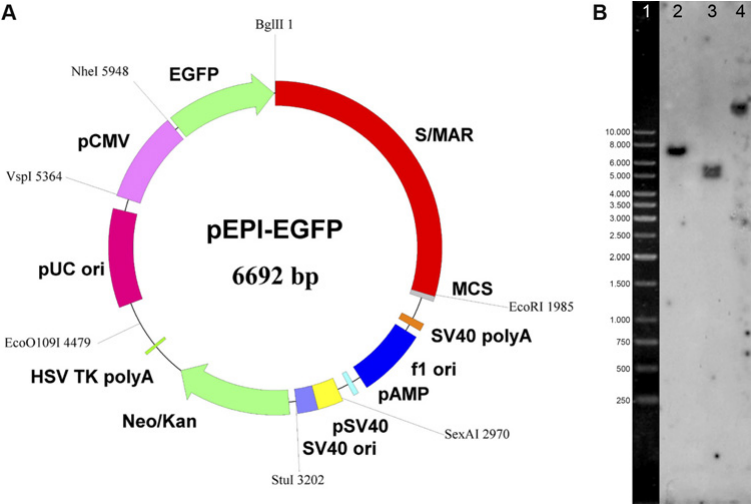
(A) Map of pEPI-EGFP. pEPI-EGFP derives from the commercial plasmid pGFP-C1 (Clontech). An S/MAR sequence, obtained from the human IFN β-gene, was cloned into the multiple cloning site (MCS) of pGFP-C1 [7] resulting in the vector pEPI-1. The GFP gene was substituted by the enhanced version EGFP in pEPI-EGFP. The Neo/Kan gene is driven by dual promoters to confer kanamycin resistance in bacteria and G418 resistance in mammalian cells. BglII and EcoRI restriction sites are indicated. SV40, simian virus 40; ori, origin of replication; HSV, herpes simplex virus. (B) Southern blot analysis of extra-chromosomal DNA and integrated vector copies using pEPI as a probe. Lane1: 1-Kbp Ladder (O'Gene Ruler; Fermentas, St. Leon-Rot, Germany). Lane2: free extra-chromosomal pEPI DNA isolated from CHO cells transfected with supercoiled plasmid DNA, linearized by digestion with BglII demonstrating the episomal state of the vector. Lane3 and 4: DNA isolated from CHO cells transfected with linearized plasmid DNA. In this case the vector integrates at random sites into the genome and can become rearranged. DNA was digested with BglII.

Here we describe how this can be achieved using the autonomously replicating vector pEPI (Figure 1A), which has been developed as an extra-chromosomal model system to study chromatin function [5-9]. In this system, episome function depends on a transcription unit linked to an efficient S/MAR (nuclear scaffold/matrix attachment region) element [5,6,8-11]. This vector can be established in proliferating cells, maintained at low copy number (2–15/cell) in all cell lines tested to date, including primary cells and animal systems [12,13]. It replicates once per cell cycle during early S-phase and is mitotically stable in the absence of selection over hundreds of generations [7,8]. The episome pEPI interacts with the prominent 'nuclear matrix' protein SAF-A [11] and an association of the vector with metaphase chromosomes has been noted [5].

One limitation of episomally replicating vectors, including those based on viral genomes, is that their establishment is inefficient, with only 1–5% of transiently transfected cells giving rise to clones that establish stable expression and propagation of the extra-chromosomal replicon [14]. Studies with pEPI have shown that once the vector has established in a cell line the extra-chromosomal DNA circles are maintained with high efficiency in the absence of selection [7,8]. Remarkably, these replicons have a mitotic stability similar to that observed for mammalian artificial chromosomes [15], even though centromeric elements are not present. The molecular mechanisms responsible for stable maintenance and propagation of pEPI in established clones is clearly based on epigenetic factors, which evolve stochastically at some low, but predictable frequency in transfected cells. Intuitively, the key phenotype must be related to mechanisms that determine the interaction of the extra-chromosomal DNA molecules with functional nuclear compartments, specifically sites of RNA and DNA synthesis.

As DNA synthesis occurs during only part of the cell cycle it is likely that key features involved in stable maintenance and propagation of extra-chromosomal replicons will be linked to RNA synthesis. During transient expression, plasmid borne genes are known to interact with active transcription factories at the nucleoskeleton, and detailed studies of the anatomy of the interactions have shown that contacts between the transcription sites and ectopic DNA are transient and dynamic – both within promoters and transcribed regions [16]. The dynamic properties of transcription-based interactions give rise to a concept of a transcript cycle, which has also been described for endogenous genes [17]. Crucially, this predicts that genes must be dynamic in order to interact with active transcription sites, suggesting that when the spatial organization of a gene is not constrained by its chromosomal context, as in an episome, it may diffuse away from the active site and become silenced. The resulting defects in gene expression would compromise the efficiency of DNA synthesis [18], with a resulting decrease in copy number of the extra-chromosomal replicons.

The molecular mechanisms that underlie the establishment of autonomous replicons in long-term mammalian cell culture are unknown, despite the potential importance of ectopic gene expression in biomedicine [19]. Towards understanding this mechanism, we have performed a detailed study of the interaction of an extra-chromosomal replicon, pEPI, with the major nuclear compartments of CHO cells. We also show that once the extra-chromosomal DNA is stably established in cell lines the ectopic DNA is efficiently maintained in the active chromatin and associates uniquely with the most active nuclear domains and early replicating chromosomal sequences. The extra-chromosomal DNA molecules are attached to chromosomes during mitosis and crucially demonstrate a high frequency of association of vector DNA molecules to equivalent positions on sister chromatids. These observations provide the first insight into a molecular mechanism that defines efficient segregation of a low copy number autonomous replicon to daughter cells during long-term cell culture.

## Results

In this study we followed the establishment and nuclear fate of the autonomous replicon pEPI-1 and its integrating counterpart. It has been shown before that vectors in which either the S/MAR or the transcription unit were deleted or in which transcription terminates upstream of the S/MAR integrate. In addition, any linearized pEPI molecule is prone to integration. The copy number of all these integrated vector molecules was estimated to be between 1 and 2 [6,9]. In contrast, integration of the supercoiled pEPI molecule is a rare event not detectable by Southern or PCR analysis of genomic DNA [6,8,9]. To analyze vector behavior after various time intervals after selection CHO cells were either transfected with pEPI in its supercoiled form or after linearization with BglII.

Cells were then selected for 10 days with G418 and grown for an additional two weeks in the absence of selection. After this time, all cells (over 99%) transfected with the supercoiled vector that survived selection showed stable expression of the GFP reporter gene. In contrast, in cells transfected with the linearized plasmid and cultured under identical conditions GFP expression was undetected in 99% of cells. During vector integration, the loss of reporter gene expression correlates with epigenetic silencing by promoter methylation [10]. In a very simplistic way, these observations provide a means of comparing the behavior of identical genetic elements under conditions where gene expression is either maintained or suppressed.

**Figure 2:**
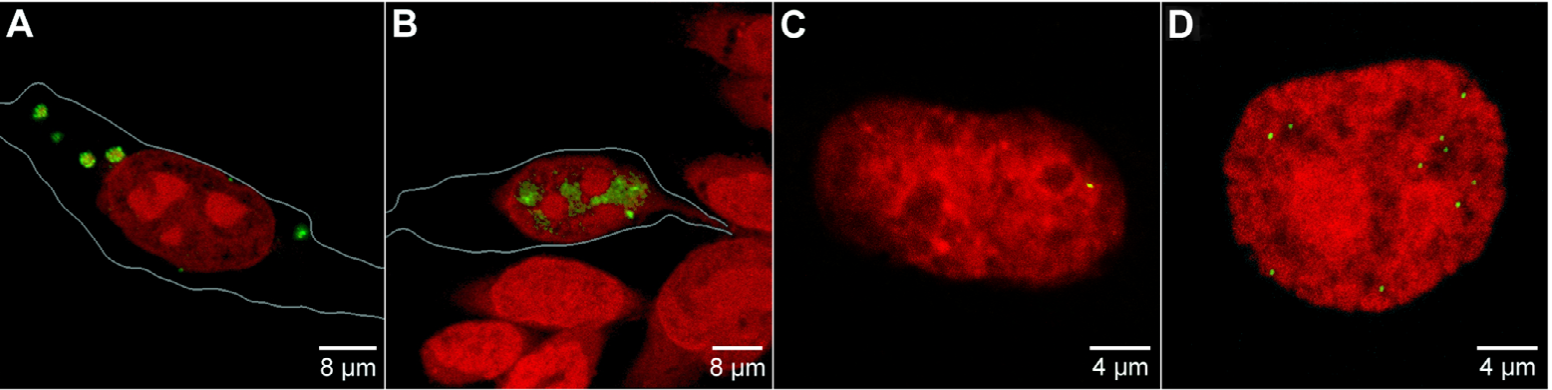
**Establishment of an autonomous replicon**. Transfection of CHO cells with pEPI or linearized vectors was performed using the lipid-based transfection reagent FuGENE 6 (Roche). Subsequently the establishment of the episome in the cell nucleus was monitored 6 h (A), 24 h (B) and after a selection period of 10 days (C, D) post transfection. The episome (green) was visualized by pEPI FISH. To-Pro-3 was used for DNA counterstaining (red) in (A-D). Maximum intensity projections were rendered from a set of 5 mid serial sections. The cellular contour was outlined with a white line in (A, B). (A) Vesicles containing numerous vector molecules occurred within the cytoplasm 6 h post transfection, whereas no signals were observed within the nucleus. (B) A strong intranuclear pEPI signal indicating a large number of vector molecules was observed 12 h post transfection, while cytoplasmic localization of pEPI was no longer observed. (C) In the majority of cases, cells transfected with the linearized vectors displayed FISH signals consistent with integration of the vector at a single chromosomal locus (D) Solely a limited number of distinct pEPI signals was observed after a selection period of 10 days suggesting that only a minor portion of the episome is stably established.

Following transfection, fluorescence in situ hybridization (FISH) was used at different time points to follow the uptake and establishment of the vector DNA (Figure 2). At 6 h post-transfection, using FuGENE6, most of the vector DNA was found in the cytoplasm (Figure 2A). After 24 h, the intensity of FISH signals was reduced and surviving vector sequences were present within the nucleus (Figure 2B). However, despite the efficient transfection in this experiment, during selection, only 0.2–0.5% of cells transfected with the linearized vector and 1–5% of cells transfected with the supercoiled construct were able to survive to generate established cell clones. In the majority of cases, cells transfected with the linearized vectors displayed FISH signals consistent with integration of the vector at a single chromosomal locus (Figure 2C). Cells transfected with the supercoiled plasmid, in contrast, displayed multiple – between 4 and 12 in this experiment – dispersed FISH signals of similar intensity (Figure 2D, see also Additional file 1). Integration is a rare event in mammalian cells, so the low efficiency with which clones can be established with integrating constructs can be easily explained. However, the process that leads only a small percentage of cells transfected with the supercoiled vector to generate established clones, in which extra-chromosomal vectors are efficiently maintained, is more difficult to understand.

### The nuclear distribution of extra-chromosomal replicons

**Figure 3:**
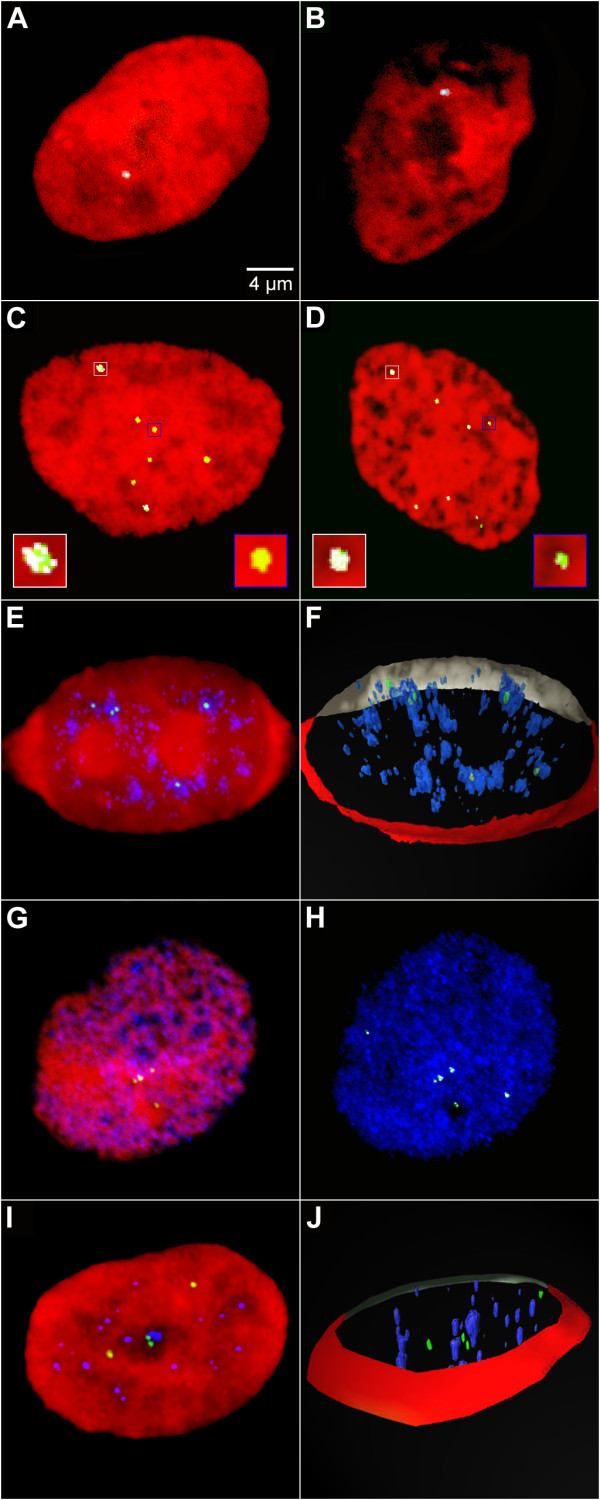
**Localization of the episome in interphase nuclei**. The qualitative co-localization of the episome with subnuclear structures in interphase nuclei was analyzed using pEPI FISH (A-J). In some experiments pEPI FISH was used in combination with immunofluorescence techniques to analyse the co-localization of pEPI with subnuclear marker proteins (E-J). Co-localizing voxels of two channels being analyzed were highlighted in white color in (A, B, C, D, H). The episome (green) was visualized by pEPI FISH. To-Pro-3 was used for DNA counterstaining (red) in (A, B, C, D, E, G, I). Maximum intensity projections were rendered from a set of 5 mid serial sections (A, B, C, D, E, G, H, I). For further details see Additional file 2. (A, B) Co-localization analyses showed that cells transfected with the linearized vectors displayed FISH signals consistent with integration of the vector at a single chromosomal locus as the vectors completely co-localized with domains containing condensed chromatin. Equal results were obtained when hyper-condensation of chromatin was induced prior to fixation (B). (C, D) Analyses of single pEPI signals revealed that the vector did not (green) or at most incompletely (white/green) colocalize to condensed chromatin regions (red). Insets are 400% magnifications of white or blue framed sectors in C and D. Such representative pEPI signals consist of green voxels (negative co-localization with condensed chromatin) or are composed of white and green voxels (incomplete co-localization with condensed chromatin). Negative or incomplete co-localization suggests that the episome is localized within the IC or at perichromatin regions bordering the IC at condensed chromatin surfaces. Equal results were obtained when hyper-condensation of chromatin was induced prior to fixation (B). (For selected light optical sections corresponding to the nuclei displayed in C and D as maximum intensity projections see the Additional file 2.) (E, F) Co-localization analyses of the episome (green) and SC35 (blue) showed that the episome occured co-localized or in close proximity to nuclear speckles, a structure which is found within the IC. (F) A 3D reconstruction was rendered from the same nucleus as displayed in (E). (G, H) Nuclear localization of the episome (green) and histone3 acetylated at lysines 9/14 (H3 acetyl-K9/K14) (blue). The nuclear counterstain (red) channel was hidden in (H) to facilitate co-localization analysis of the episome and H3 acetyl-K9/K14 showing that the episome occurred co-localized or in close proximity to sites of active transcription. (I, J) Co-localization analyses of the episome (green) and histone3 trimethylated at lysine9 (H3 trimethyl-K9) (blue) showed that the episome did not co-localize with such sites. (J) A 3D reconstruction was rendered from the same nucleus as displayed in (I). Bar in (A) is representative for (A-J).

To find a rational explanation for this phenomenon a mixed population of G418 resistant cells transfected with supercoiled pEPI was propagated for another two weeks in the absence of selection and the distribution of the vector molecules compared to well-characterized nuclear structures. The fate of the vector in cells transfected with the supercoiled vector was verified by Southern analysis and rescue experiments (Figure 1B and data not shown). Strikingly, FISH experiments and subsequent bioinformatical co-localization analyses on interphase nuclei showed that over 99% (275 out of 276 scored; Additional file 1) of the episomal vector molecules were localized in the interchromatin compartment (IC) or in perichromatin regions adjacent to condensed chromatin domains [1,20] (Figure 3C, D, see also Additional file 1 and Additional file 2). The IC is defined as a three-dimensionally expanding nuclear domain devoid of chromatin and carrying nuclear bodies and nuclear speckles [21]. The protein SC35 is a characteristic protein of nuclear speckles. These structures were therefore visualized by immunofluorescence using an anti-SC35 antibody and co-localization analyses were performed as described above. A 3D reconstruction is shown in Figure 3F. Notably, at the light microscopic level almost all episomes localized to the borders or in very close proximity to nuclear speckles (Figure 3E, F) and the same localization and distribution was seen throughout G1-, S- and G2-phase of the cell cycle. In contrast, FISH analyses on cells transfected with the linearized vector, and in which transgene expression was silenced in the majority of cells, showed hybridization signals in dense chromatin regions. Out of 50 cells analyzed, no examples of association with the interchromatin compartment were seen (Figure 3A,B). This demonstrates that the transcriptionally active extra-chromosomal genes maintain a close association with the nuclear compartments that contains the most highly expressed endogenous genes [22,23], while integrated genes, which tend to be silenced, are excluded from this compartment.

Post-translation modifications in chromatin, such as N-terminal acetylation of histone H3, also correlate with transcriptional status [24]. It is known that an active transcription unit linked to a S/MAR are required to maintain the episomal status of the pEPI vector [9]. We therefore analyzed the localization of the vector in relation to the nuclear distribution of histone3 acetylated at lysines9 and 14 (H3 acetyl-K9/K14; open chromatin). For comparison, we also analyzed the vector localization and the nuclear distribution of histone3 trimethylated at lysine9 (H3 trimethyl-K9; condensed chromatin), which is a marker for genes that are not transcribed [25]. In almost all cases, pEPI was at least partly co-localized with nuclear domains containing H3 acetyl-K9/K14 (Figure 3G, H) whereas no association with H3 trimethyl-K9-rich domains was seen (Figure 3I, J).

**Figure 4:**
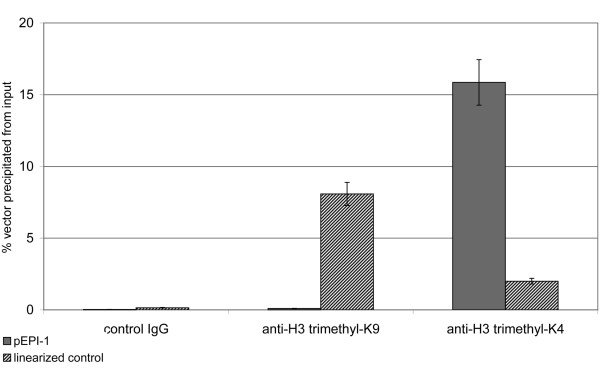
**Quantitative real time PCR analyses of ChIP experiments**. ChIP experiments were performed using antibodies targeted to H3 trimethyl-K9 (condensed/inactive chromatin: mid columns), H3 trimethyl-K4 (open/active chromatin: right handed columns) or using a control IgG (left handed columns). The percentage of either pEPI-1 (gray columns) or the linearized control vector (striped columns) precipitated from input was quantified using real time PCR. Since no significant amount of the vector molecules was pulled down with the control IgG, it could be clearly demonstrated that about 30 times more vector molecules were precipitated using the anti-H3 trimethyl-K9 antibody in a precipitate from cells containing the integrated vector compared to cells containing the vector in its episomal form. When the same chromatin preparations were precipitated with an antibody directed against H3 trimethyl-K4 over 20 times more vector molecules were obtained from cells containing the episome compared to cells containing the integrated vector.

The observation that an established pEPI molecule is associated and organized in an active chromatin conformation was further supported by chromatin immunoprecipitation (ChIP) experiments. Chromatin was isolated from a mixed cell population containing the vectors either in its episomal status or integrated into the genome. Antibodies directed against various histone variants were added and the amount of vector molecules present in the precipitate was quantified by real time PCR using primers to amplify various regions (S/MAR, eGFP) of the vector. As in our microscopic analyses H3 trimethy-K9 was used as a marker for inactive chromatin. Unfortunately the antibody directed against H3 acetyl-K9/K14 used in our microscopic analyses as a marker for active chromatin was not suitable for ChIP analyses. Therefore an antibody directed against H3 trimethyl-K4 which is also a marker for transcriptionally active chromatin [26,27] was used. Figure 4 shows the results of these ChIP analyses using primers directed against eGFP, a similar picture was obtained using primers directed against the S/MAR. When chromatin concentration of the two cell population was adjusted to the same number of vector molecules present and precipitated with an antibody directed against H3 trimethyl-K9 30 times more vector molecules were found in a precipitate from cells containing the integrated vector compared to cells containing the vector in its episomal form. When the same chromatin preparations were precipitated with an antibody directed against H3 trimethyl-K4 over 20 times more vector molecules were obtained from cells containing the episome compared to cells containing the integrated vector. This clearly demonstrates that the episome co-precipitates with an active chromatin conformation while the majority of integrated vector molecules are found to be associated with inactive chromatin.

### Extra-chromosomal DNA molecules associate with chromosomes to ensure efficient mitotic segregation

**Figure 5:**
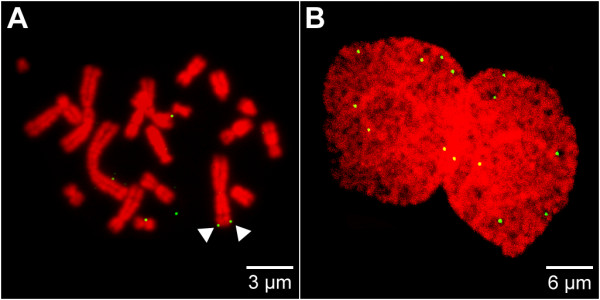
**Association of the episome with metaphase chromosomes**. The localization of the episome was studied by FISH on spreads of metaphase chromosomes (A), and the equal distribution of vector molecules was monitored in postmitotic nuclei of dividing cells (B). The episome (green) was visualized by pEPI FISH. To-Pro-3 was used for DNA counterstaining (red). A maximum intensity projection was rendered from a set of 5 mid serial sections in (B). Arrows in (A) indicate a pEPI signal pair, each signal localized on a sister chromatid. For further details see Additional file 4.

Random distribution of a low copy number (5–10 copies/cell) vector would imply that during each division a significant percentage of cells should lose the replicon – stochastically about one third of the daughter cells would have lost the vector molecules after two generations. Instead, a mitotic stability of over 0.98 has been observed with the vector system used in this study [6,8,10]. The possibility that vector molecules might associate preferentially with a limited number of specific sites on host chromosomes could provide a mechanistic explanation for both the low and stable copy number and high mitotic stability in established clones. To test this individual clones were isolated and the location of vector molecules bound to host chromosomes analyzed in over 50 metaphase plates (details in Additional file 3 and Additional file 4). A typical FISH analysis on a CHO metaphase spread is shown in Figure 5A. In all spreads, vector molecules were mostly associated with chromatids, though the association appears to be weak as some vector molecules associated with metaphase plates were detached, presumably as a result of shear forces generated during spreading (Figure 5A). We estimate that ~50% of episomes are lost during spreading – a typical metaphase plate (4N DNA content) had on average 5.8 vector molecules (312/54 metaphase cells) while, in the same clone, nuclei in very early G1 (2N DNA content) had 5.9 pEPI molecules (297/50 early G1 nuclei) (Additional file 3 and Additional file 4).

Notably, in the individual cells of established clones vector molecules were not associated with specific chromosomal positions (Additional file 3 and Additional file 4). Instead, the episomes appeared to be randomly associated with DAPI/To-Pro-3-light staining regions, with the frequency of vector binding to a particular chromosome being proportional to the size of the chromosome. Despite the apparently random nature of these interactions, it was immediately obvious that in those spreads where single chromosomes had two associated episomes an unexpectedly high proportion had the two epsiomes associated with identical positions on the sister chromatids (Figure 5). In the clone analyzed in Table S-1 (Additional file 1), 35% (110/312) of FISH signals were from chromosomes with two episomes. Of these, 53% (58/110) had the two signals associated with identical positions on the sister chromatids. If such paired episomes are normally preserved throughout mitosis – remember 50% of episomes are lost during spreading – this provides a clear mechanism to account for the efficiency with which episomes are maintained in established cell clones.

To extend this observation we next analyzed the distribution of vector molecules in daughter cell pairs, following mitosis (Figure 5B and Additional file 5). Remarkably, when 50 early G1-phase nuclei were analyzed – as 25 daughter pairs – we found that the number of episomes in the sisters was identical, or differed by only 1, in 84% (42/50) of cases. An example of the distribution of vector molecules in two early G1 sister nuclei is shown in Figure 5B; in this example, each of the two nuclei contains 7 copies. Interestingly, as in this case, the vector molecules were usually arrayed with striking mirror symmetry, which is consistent with what would be expected if the episomes remained attached to sister chromatids throughout mitosis.

### Extra-chromosomal replicons maintain stable associations with early replicating DNA foci

**Figure 6:**
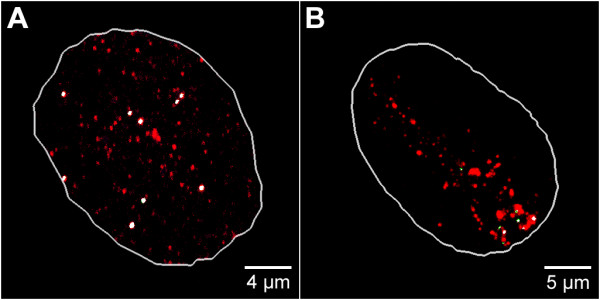
**Co-localization of the episome with early replication foci**. The qualitative co-localization of the episome and replication foci was analyzed using pEPI FISH and BrdU pulse labeling. Maximum intensity projections were rendered from a set of serial sections. The nuclear contour was outlined with a white line. (A) Replication foci were labeled during early S-phase by incorporation of BrdU (1 h) into newly synthesized DNA. Subsequently nuclei were fixed and BrdU (red) was visualized by antibody staining. The episome (green) was visualized by pEPI FISH. Co-localizing voxels were highlighted in white color. (B) Alternatively nascent DNA was pulse-labeled with BrdU for 1 h during early S-phase, fixing and subsequent immunolocalization was done following cell division. The episome (green) was visualized by pEPI FISH. Co-localizing voxels were highlighted in white color.

As stable maintenance of the episome in established clones requires both efficient replication and segregation, we also attempted to define the extent of interaction between episomes and early replicating DNA foci, which contain the most highly expressed chromosomal genes [1]. When cells were pulse-labeled with BrdU the episomal replicons, identified by FISH, were shown to co-localize with DNA foci that displayed the classical size, shape and distribution of those that are labeled during early S-phase replication (Figure 6A). As the vector DNA is known to replicate once per cell cycle during early S-phase [8], this observation suggests that the efficiency of replication is linked to the association of the vector with early replicating host cell sequences. Moreover, a clear association with the early replicating DNA foci is maintained throughout the cell cycle, and can even be seen following mitosis, when the majority of the vector molecules still co-localized with BrdU-labeled foci (Figure 6B). This demonstrates that vector molecules are able to associate with high efficiency to early replicating DNA and raises the possibility that the episomes can engage in stable interactions with the active chromatin domains throughout the cell cycle.

## Discussion

In previous studies, we have described the structure of an extra-chromosomal gene delivery system, which is able to establish stable cell clones and provide prolonged gene expression over at least 100 cell division cycles and in the absence of selection pressure [19]. Established cell clones have a stable and low vector copy number. However, the efficiency of vector establishment in the cells is low, and the molecular mechanisms that support efficient episome maintenance are unknown.

### Extra-chromosomal replicons form stable associations with active nuclear domains throughout the cell cycle

In this study, we set out to develop a model to explain how mammalian cells could maintain extra-chromosomal episomes with high efficiency. The key to our approach was to define in detail how the extra-chromosomal replicons interact with major nuclear compartments. We have shown that episomes within established clones are associated with the most highly transcribed nuclear domains (Figure 3) and early replicating chromatin (Figure 6). These associations are clearly stable over time, and crucially appear to persist throughout mitosis, where episomes remain attached to peripheral domains of condensed chromosomes (Figure 5B).

Following mitosis, extra-chromosomal DNA molecules were shown to be segregated to sister nuclei with remarkable efficiency and uniformity (Figures 5 and 6 and Additional file 5).

To determine whether the vectors associate with specific chromosomal sites we analyzed their localization on metaphase spreads. In these analyses no vector binding to specific chromosomal regions could be found. The interaction of the vector molecules with the chromatids seemed to be weak and it could be estimated that about 50% of the molecules are lost during spreading. Moreover in these experiments it is not absolutely possible to discriminate between a specific association of plasmids to chromosomes in vivo and a non-specific trapping of vector molecules during the spreading procedure. However, the frequency of observed pair-wise association and the observed association of all vector molecules with DAPI/To-Pro-3 light staining regions of the chromatids argues against non-specific vector trapping.

These observation suggests that the DNA molecules maintain a stable association with early replicating DNA foci, and that replicating in synchrony with these foci provides a mechanism that allows the sister episomes to be segregated with their associated sister chromatids. This idea is also supported by the pseudo-symmetrical distribution of episomes in daughter nuclei following mitosis (Figure 5B), which is similar to the distribution of chromosomes in daughter cells [28]. These observations provide the first mechanistic insight into how an episome might achieve stable establishment and propagation in proliferating cells.

### Epigenetic changes drive establishment of cell clones with stably maintained extra-chromosomal replicons

The key genetic elements required to develop cell clones that propagate extra-chromosomal DNA molecules are an efficient gene expression cassette linked to a strong S/MAR element [6,9,10]. However, as these elements drive the establishment of stable clones in <5% of cells that contain the basic replicon it is clear that the structure of the genetic components is not the only critical feature. The obvious corollary of this is that during the establishment phase epigenetic changes evolve to yield an extra-chromosomal replicon, with a specific chromatin status, which ensures efficient gene expression and early replication. Crucially, this epigenetic programming must also ensure that the ectopic replicon maintains a stable association with the active nuclear domains. The mechanism that defines this behavior is unknown. However, as episomes can be recovered from established clones and shown to be genetically unaltered we can be sure that the critical events result from epigenetic changes that arise stochastically in transfected cells [7]. Strong support for this idea comes from recent experiments in which pEPI was used to genetically modify pigs. When the vector was introduced by sperm-mediated gene transfer into the egg, which is known to be an efficient environment for epigenetic re-programming, the vector was found to be maintained in an extra-chromosomal form and actively expressed in a range of tissues in ~70% of fetuses [12]. Clearly, in this model system, the extra-chromosomal replicons were maintained with very high efficiency during all division cycles.

## Conclusion

For all the reasons discussed above the behavior of a minimal replicon described in the present study provides a new model system for the analyses of the epigenetic regulation of nuclear function in higher eukaryotes. The results of this study support the idea that the precision of these functions is strongly influenced by higher nuclear architecture. Furthermore our findings have relevance for the rational design of episomal vectors to be used for genetic modification of cells: in order to improve such constructs with respect to efficiency elements have to be identified which ensure that such constructs reach regions of the nucleus favorable for replication and transcription.

## Methods

### Cell culture, transfection and synchronization

CHO cells were cultured in Ham's F12 medium supplemented with 10% fetal calf serum (FCS). Transfections were performed using the lipid-based transfection reagent FuGENE6 (Roche) as recommended by the manufacturer. After 48 h cells were selected with 500 μg/ml G418. Stable clones were isolated after 2 weeks. Individual clones were isolated and cultured with 250 μg/ml G418 for 3 to 4 weeks.

Cells were synchronized using isoleucine-free medium with 0.5% dialyzed FCS for 48 h (G_0-_phase arrest). Subsequently cells were washed and released for 9 h into complete medium containing an excess of thymidine for arrest at the G1/S-phase transition [29].

### Isolation of DNA, Southern analysis and quantitative PCR

Isolation of total cellular DNA or DNA from the Hirt extract, Southern analyses and real-time PCR to determine the vectors copy number were performed as described before [7,8].

### Metaphase spreads

10^8 ^cells were arrested in mitosis by incubating them with 0.2 μg/ml colcemid (Biochrom) for 3 h at 37°C. After mitotic shake off the cells were carefully resuspended in 0,56% KCl solution for 15 minutes at 37°C. After centrifugation at 1000 rpm for 10 minutes nuclei were fixed in ice-cold methanol:glacial acetic acid (3:1) for 30 minutes at -20°C and collected again by centrifugation at 4°C. Fixation was repeated for at least three times. Nuclei were then resuspended in 1 ml fixative and dropped on 55°C pre-heated microscopic slides under a moist environment. Slides were dehydrated in a series of 70%, 90%, 100% ethanol for 5 minutes each and kept at room temperature for another day. Slides were then incubated with 0.005% pepsin in 0.01 M HCl for 5 minutes at 37°C, washed in PBS and dehydrated again in a ethanol series.

### Replication labeling

Prior to replication labeling cells were synchronized and arrested in early S-phase using 1 μg/ml aphidicolin (Sigma) added to HAMS-F12 medium and incubation for 18 h. Subsequently cells were washed briefly with pre-warmed PBS, and then incubated in HAMS-F12 medium containing 50 μM 5-bromo-2'-deoxyuridine (BrdU) for 1 h (early S-phase). Alternatively after washing a 3 h chase was allowed prior to BrdU-labeling for 1 h (mid S-phase). Subsequently cells were fixed using 4% paraformaldehyde.

### Chromatin hyper-condensation

Hyper-condensed chromatin (HCC) formation was induced for some experiments by incubating cells in a hyper-osmolar medium at osmolarities above 380 mOsm/l as described elsewhere [30]. Since processes of chromatin condensation and decondensation started immediately when osmolarities changed, washing steps in physiological salt solutions, such as 1 × PBS (290 mOsm/l) were strictly avoided prior to the fixation of cells.

### Fluorescence in situ hybridization of pEPI (pEPI FISH)

Probes for pEPI FISH were labeled with digoxigenin-dUTP (DIG-dUTP). A pEPI construct which does not contain the S/MAR sequences was used for probe labeling with DIG-dUTP by PCR, or alternatively by nick translation. Probes and salmon sperm competitor DNA were dissolved in hybridization mixture (50% formamide, 10% dextran sulfate, 2 × SSC).

Cells were fixed as described above. Nuclei were permeabilized with 0.5% Triton-X100 in PBS for 20 minutes, followed by incubation with 0.1N HCl for 5 minutes at room temperature. Nuclei were subsequently treated with 200 μg/ml RNaseA for 30 minutes at 37°C. Preparations were then equilibrated in 50% formamide in 2 × SSC for 7 days at 4°C. Subsequently DNA was denatured with 70% formamide in 2 × SSC, pH7.4 for 3 minutes at 72°C. The probe was denatured separately in a boiling water bath for 10 minutes, briefly chilled on ice and subsequently loaded onto a coverslip with immobilized and fixed cells. Hybridization was performed overnight at 37°C followed by post hybridization washes in 2 × SSC at 37°C and 0.1 × SSC at 60°C. Blocking was done in 4 × SSCT (4 × SSC, 0.2% Tween) containing 4% BSA for 20 minutes at room temperature. Antibodies were diluted in blocking solution as described below. Between application of primary and secondary antibodies washing steps were performed in 4 × SSCT for 15 minutes. Incubations with all antibodies were sequentially performed for 1.5 h at 37°C. For detection of digoxigenin-labeled probes rabbit anti-digoxigenin antibody (Sigma, catalogue no. D7782) was used as primary layer, goat anti-rabbit conjugated with Cy3 (Jackson ImmunoResearch Laboratories, catalogue no. 111-166-003) was used as secondary layer. Nuclei were counterstained with 0.1 μg/ml 4',6-diamidino-2-phenyl-indole (DAPI) (Sigma) and 1 μM To-Pro-3 (Molecular Probes) in PBS and subsequently washed with PBS. Preparation were mounted in Vectashield antifade medium (Vector Laboratories) and sealed with nail polish.

### Immunofluorescence

In some cases splicing factor SC35, histone3 acetylated at lysines9 and 14 (H3 acetyl-K9/K14), or histone 3 trimethylated at lysine9 (H3 trimethyl-K9) were detected following pEPI FISH. Prior to detection nuclei were equilibrated in PBS for 20 minutes at room temperature. Blocking was done in PBST (PBS, 0.1% Triton-X100) containing 4% BSA for 20 minutes at room temperature. Antibodies were diluted in blocking solution. Between application of primary and secondary antibodies washing steps were performed in PBST for 20 minutes. Incubations with all antibodies were performed for 1.5 h at 37°C. SC35 was detected with monoclonal mouse anti-SC35 antibody (Sigma, catalogue no. S4045) and then with goat anti-mouse antibody conjugated with AlexaFluor488 (Molecular Probes, catalogue no. A-21121). H3 acetyl-K9/K14 was detected with polyclonal rabbit anti-H3 acetyl-K9/K14 antibody (Santa Cruz, catalogue no. sc-8655-R) and then with goat anti-rabbit antibody conjugated with AlexaFluor488 (Molecular Probes, catalogue no. A-11054). H3 trimethyl-K9 was detected with polyclonal rabbit anti-H3 trimethyl-K9 antibody (Upstate, catalogue no. 07-442) and then with goat anti-rabbit antibody conjugated with AlexaFluor488 (Molecular Probes, catalogue no. A-11054). Nuclei were counterstained; preparations were mounted and sealed as described above.

### Immunodetection of BrdU

In some cases BrdU incorporated into nascent DNA was detected following pEPI FISH. Prior to detection nuclei were equilibrated in PBS for 20 minutes at room temperature. Blocking was done in PBST (PBS, 0.1% Triton-X100) containing 4% BSA for 20 minutes at room temperature. Antibodies were diluted in blocking solution. Between application of primary and secondary antibodies washing steps were performed in PBS for 20 minutes. Incubations with all antibodies were performed for 1.5 h at 37°C. BrdU was detected with a monoclonal mouse anti-BrdU antibody (Sigma, clone BU 33, catalogue no. B 2531) and then with goat anti-mouse antibody conjugated with AlexaFluor488 (Molecular Probes, catalogue no. A-21121). Nuclei were counterstained; preparations were mounted and sealed as described above.

### Confocal laser scanning microscopy (CLSM)

Nuclei were analyzed by CLSM. Acquisition of serial sections was done with a Leica TCP SP confocal laser scanning microscope (Leica Microsystems, Mannheim) equipped with an oil immersion PlanApochromat100/1.4NA objective lens. Fluorochromes were visualized with an argon laser with excitation wavelengths of 488 nm for AlexaFluor488 and 514 nm for Cy3, and with a helium-neon laser with excitation wavelength of 633 nm for To-Pro-3. The chromatic shift was measured using multi-labeled microbeads. Fluorochrome images were scanned sequentially generating 8-bit grayscale images. Image resolution was 512 × 512 pixels with a pixel size ranging from 195 to 49 nm depending on the selected zoom factor. The axial distance between light optical serial sections was 250 nm. To obtain an improved signal-to-noise ratio each section image was averaged from four successive scans. The 8-bit grayscale single channel images were overlaid to an RGB image assigning a false color to each channel and then assembled into tables using open source software ImageJ (Rasband, W.S., ImageJ, U. S. National Institutes of Health, Bethesda, Maryland, USA) and Adobe Photoshop 7.0 software. 3D reconstructions were performed by surface rendering using AMIRA software (Amira 3.1, TGS Europe).

### Co-localization analyses

Qualitative co-localization analyses were performed using the *Co-localisation Highlighter *plugin of ImageJ (Rasband, W.S., ImageJ, U. S. National Institutes of Health, Bethesda, Maryland, USA). Prior to co-localization analyses the chromatic shift was adjusted, and threshold settings for each 8-bit stack (red and green respectively) were determined using the automatic thresholding function and assigned to the input window of the *Co-localisation Highlighter *plugin. The co-localized voxels intensity ratio was set at 50%. The resulting red and green 8-bit channels were overlaid to an RGB stack. Co-localized voxels were highlighted in white color. An image was created using the *Maximum Intensity *projection type along the Z-axis.

### In vivo cross-linking

1% formaldehyde was added directly to the media and cells were incubated on a shaker at room temperature for 10 min. Subsequently 2.5 M glycin was added to stop the reaction. Cells were washed with ice-cold PBS, scrapped off and then centrifuged at 2000 rpm, 4°C. The pellet was twice resuspended in 10 ml Triton-X buffer (0.25% Triton X-100, 10 mM EDTA, 10 mM Tris/HCl (pH8.1), 10 mM NaCl, 1× CompleteMini protease inhibitor (Roche)), shaked on ice for 10 min and centrifuged at 2000 rpm, 4°C. The nuclear lysate was resuspended in SDS lysis buffer (1%SDS, 5 mM EDTA, 50 mM Tris/HCl (pH8.1), 1× CompleteMini protease inhibitor (Roche)). Chromatin was sheared to an average fragment length of 200–1200 bp by sonication on ice (Branson sonifier 250).

### Chromatin immunoprecipitation (ChIP)

Sepharose-Protein A beads (Zymed) were washed twice with PBS/0.1% TritonX-100, centrifuged for 2 min at 900 rpm, 4°C and then washed twice with TSE I buffer (0.1% SDS, 1% TritonX-100, 2 mM EDTA, 20 mM Tris/HCl (pH8,1), 150 mM NaCl). Beads were collected and equilibrated in TSE I buffer. The chromatin isolate was centrifuged at 13.000 × g for 15 min at 4°C, the supernatant was transferred to a new 15 ml vial. Chromatin was diluted with 5 ml dilution buffer (1% TritonX-100, 2 mM EDTA, 20 mM Tris/HCl (pH8.1), 150 mM NaCl, 1× CompleteMini protease inhibitor (Roche)). To preclear the supernatant was incubated with 60 μl bead suspension on a shaker for 1 h at 4°C. For blocking remaining beads were shaked on ice for 2 h with 67 μl BSA (20 mg/ml) and sonicated salmon sperm DNA (1.5 μg per 20 μl bead suspension), then centrifuged for 2 min at 3500 rpm and equilibrated in TSE I buffer at 4°C. Antibodies used for ChIP were targeted to histone 3 trimethylated at lysine 4 (H3 trimethyl-K4/Abcam, catalogue no. ab8580) or histone 3 trimethylated at lysine 9 (H3 trimethyl-K9/Upstate, catalogue no. 07-442). Rabbit anti-digoxigenin IgG (Sigma, catalogue no. D7782) was used as a control. For each ChIP 1 mg of precleared supernatant was incubated overnight on a shaker with 3 μg of antibody at 4°C in the presence of 30 μl blocked bead solution. 1 mg of the non-precipitated chromatin was saved to determine the input DNA amount. Following ChIP the immunocomplexes were centrifuged at 3500 rpm for 2 min at 4°C, resuspended in 2 ml ice-cold dilution buffer, transferred into a new vial and another 2 ml dilution buffer was added. After centrifugation the pellet was incubated twice in 10 ml ice-cold TSE I buffer for 10 min shaking, then twice in 10 ml buffer III (0.25 M LiCl, 1% Nonident P-40, 1% deoxycholate, 1 mM EDTA, 10 mM Tris/HCl (pH8.1)) and then once with 10 ml TE buffer (2 mM EDTA, 10 mM Tris/HCl (pH8.1)). The beads were resuspended in 300 μl elution buffer (1% SDS, 10 mM EDTA, 50 mM Tris/HCl (pH8.1)) and transferred to a new vial. To separate the beads from the immunocomplexes they were incubated at 65°C for 30 min and vortexed extensively every 5 min for 30 sec. Subsequently beads were centrifuged. The supernatant was transferred to a new vial and incubated at 65°C for 6 h in the presence of proteinase K to reverse the crosslinking. DNA was purified by standard phenol-chloroform extraction and subsequently precipitated with ethanol in the presence of 1 μg glycogen as a carrier. The precipitated DNA was dissolved in 50 μl H_2_O and treated with RNaseA.

### Quantitative real-time PCR

Quantitative real-time PCR analyses were performed using a Light Cycler instrument (Roche Diagnostics). For PCR reactions a ready-to-use “hot start” reaction mix (FastStart DNA MasterPLUS SYBR Green I; Roche Diagnostics) was used containing Taq-DNA polymerase and SYBR Green I fluorescent dye for real time detection of double stranded DNA. Reaction volume was 10 μl including 0.3 μM of each primer (GFP left: 5'-GTCAGTGGAGAGGGTGAAGG-3'; GFP right: 5'-TACATAACCTTCGGGCATGG-3'). PCR reactions were performed at 40 cycles using standard settings recommended by the manufacturer.

## Authors' contributions

IMS established transfected cell lines, performed molecular genetic studies and prepared samples for microscopic analyses, JP performed and evaluated microscopic analyses, SR performed ChIP analyses, TC participated in the design of this study, DAJ participated in the design of this study and wrote the manuscript, HJL designed experiments and wrote the manuscript.

## Supplementary Material

Additional file 1 Table S-1. Co-localization of pEPI episomes with the interchromatin compartment. Click here for file

Additional file 2 Figure S-1. Selected single optical sections corresponding to maximum intensity projections displayed in Figure 3C,D. Click here for file

Additional file 3 Figure S-2. Average number of episomes bound to each of the 20 chromosomes of CHO C400 cells. Click here for file

Additional file 4 Table S-2. FISH analyses of metaphase spreads of pEPI transfected CHO C400 cells. Click here for file

Additional file 5 Table S-3. Distribution of pEPI molecules to daughter nuclei after mitosis. Click here for file
