# Supplementary material for: Establishment and mitotic stability of an extra-chromosomal mammalian replicon
Source: BMC Cell Biol. 2007 Aug 6;8:33. doi: 10.1186/1471-2121-8-33 (PMC1959191; doi:10.1186/1471-2121-8-33)
Supplement: Additional file 1 — Table S-1. Co-localization of pEPI episomes with the interchromatin compartment. [file 1471-2121-8-33-S1.pdf]

<u>Additional file 1</u>: Establishment and mitotic stability of an extra-chromosomal mammalian replicon

Isa M. Stehle, Jan Postberg, Sina Rupprecht, Thomas Cremer, Dean A. Jackson and Hans J. Lipps

Co-localization with interchromatin compartment (IC)

Nuclei	Co-localization with CT	Co-localization with IC
1	0	6
2	0	11
3	1	12
4	0	9
5	0	8
6	0	3
7	0	3
8	0	15
9	0	9
10	0	13
11	0	6
12	0	6
13	0	8
14	0	8
15	0	7
16	0	4
17	0	3
18	0	4
19	0	4
20	0	6
21	0	4
22	0	7
23	0	4
24	0	3
25	0	4
26	0	3
27	0	2
28	0	6
29	0	2
30	0	4
31	0	3
32	0	2
33	0	5
34	0	2
35	0	3
36	0	3
37	0	5
38	0	3
39	0	2
40	0	5
41	0	1
42	0	4
43	0	4
44	0	2
45	0	6
46	0	10
47	0	8
48	0	8
49	0	9
50	0	6
Total	1	275
coloc. in %	0.36	99.64
pEPI molecules per IC		5.5

Table S-1 Co-localization of pEPI episomes with the interchromatin compartment.

Localization of the vector molecules in the nucleus was determined by FISH analysis and evaluated as described in Methods. Strikingly, FISH experiments on interphase nuclei show that over 99% of the vector molecules are localized in chromatin-poor regions of the nucleus, the interchromatin compartment (IC), and only 1 pEPI signal was detected within a chromosome territory (CT) (Table S-3). Furthermore, this co-localization of the vector with the IC was seen throughout G1, S and G2 phase of the cell cycle, supporting the stable association of pEPI with the nuclear domain that is highly enriched for the most active endogenous genes.
