# Supplementary material for: Establishment and mitotic stability of an extra-chromosomal mammalian replicon
Source: BMC Cell Biol. 2007 Aug 6;8:33. doi: 10.1186/1471-2121-8-33 (PMC1959191; doi:10.1186/1471-2121-8-33)
Supplement: Additional file 2 — Figure S-1. Selected single optical sections corresponding to maximum intensity projections displayed in Figure 3C,D. [file 1471-2121-8-33-S2.pdf]

<u>Additional file 2</u>: Establishment and mitotic stability of an extra-chromosomal mammalian replicon

Isa M. Stehle, Jan Postberg, Sina Rupprecht, Thomas Cremer, Dean A. Jackson and Hans J. Lipps

Localization of the episome in interphase nuclei.

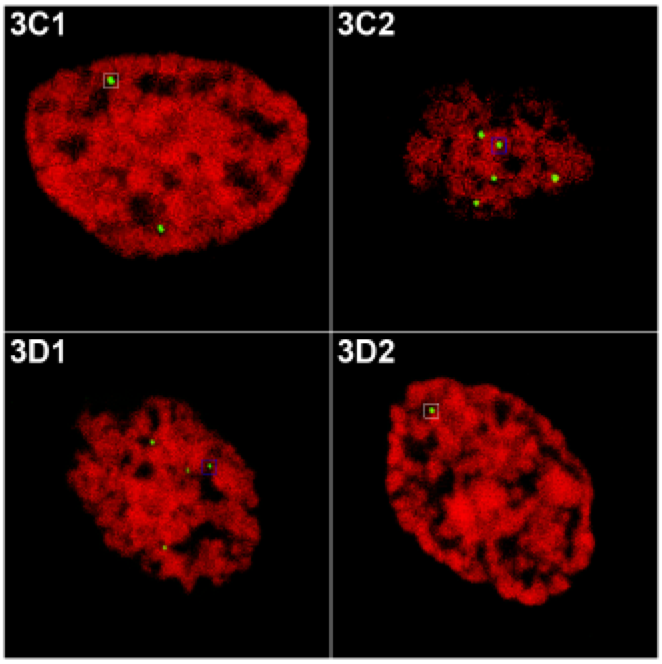

Figure S-1 Selected single optical sections corresponding to maximum intensity projections displayed in Figure 3C,D.

The vector (green) and chromatin (red) were visualized as described above. Framed sectors (white or blue) correspond to Figure 3. These single optical sections illustrate that pEPI apparently is localized within or at the borders of nuclear compartment that are devoid of dense chromatin. The results were confirmed by qualitative co-localization analyses as described in *Methods*.
