# Supplementary material for: Establishment and mitotic stability of an extra-chromosomal mammalian replicon
Source: BMC Cell Biol. 2007 Aug 6;8:33. doi: 10.1186/1471-2121-8-33 (PMC1959191; doi:10.1186/1471-2121-8-33)
Supplement: Additional file 3 — Figure S-2. Average number of episomes bound to each of the 20 chromosomes of CHO C400 cells. [file 1471-2121-8-33-S3.pdf]

<u>Additional file 3</u>: Establishment and mitotic stability of an extra-chromosomal mammalian replicon

Isa M. Stehle, Jan Postberg, Sina Rupprecht, Thomas Cremer, Dean A. Jackson and Hans J. Lipps

Metaphase spreads

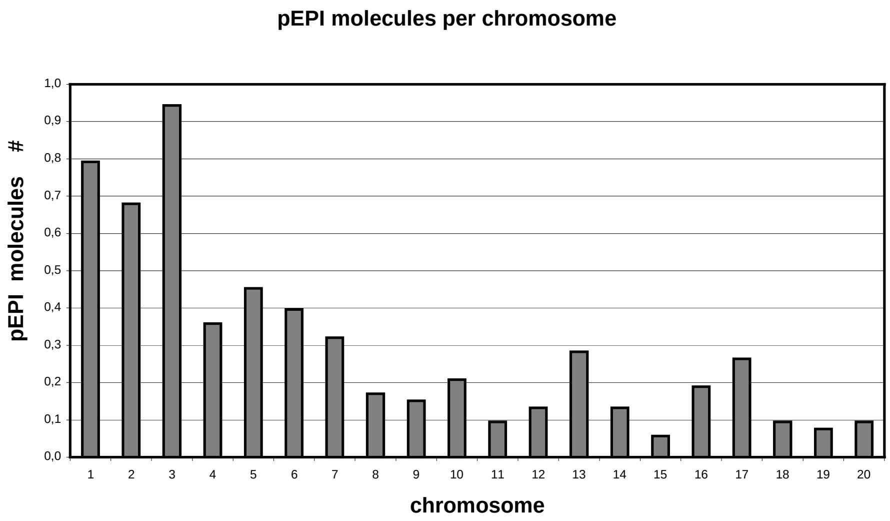

Figure S-2 Average number of episomes bound to each of the 20 chromosomes of CHO C400 cells.

FISH analyses on metaphase chromosomes were performed using pEPI as a probe. 50 metaphase plates were evaluated and the number of vector molecules attached to each chromosome determined. Chromosomes were identified by size and shape (Table S-1). A typical metaphase has in average 5.8 molecules, but vector molecules were not associated with the same chromosomes in individual cells of established clones, nor did they bind to preferred chromosomal sites (Figure S-1). This supports a mass dependent binding of the episomes to the chromosomes, with the frequency of vector binding to a particular chromosome being proportional to the size of the chromosome. About 20% of all vector molecules detected, were attached to both chromatids of a chromatid pair at the same position (Table S-1).
