# Supplementary material for: Establishment and mitotic stability of an extra-chromosomal mammalian replicon
Source: BMC Cell Biol. 2007 Aug 6;8:33. doi: 10.1186/1471-2121-8-33 (PMC1959191; doi:10.1186/1471-2121-8-33)
Supplement: Additional file 4 — Table S-2. FISH analyses of metaphase spreads of pEPI transfected CHO C400 cells. [file 1471-2121-8-33-S4.pdf]

<u>Additional file 4</u>: **Establishment and mitotic stability of an extra-chromosomal mammalian replicon**

Isa M. Stehle, Jan Postberg, Sina Rupprecht, Thomas Cremer, Dean A. Jackson and Hans J. Lipps

Metaphase spreads	Chromsomes of CHO C400 cells 1	2	3	4	5	6	7	8	9	10	11	12	13	14	15	16	17	18	19	20	pEPIs per cell
1		1	*2		1	1		1				1									7
2		1	1		1		2			*2			2			1					10
3	1	1	1													1				1	5
4	1		*2	2		1					1				1						8
5		1			*2				*2												5
6	1	1	2			1				1			1					1			8
7	2		*2		1		1		1												7
8	*2			1	1		1	1	1				1								8
9		2		2	1		1		1											1	8
11	1	1	*2							1											5
13	*2		*2	1	1	2	1		1				1		1						12
14	2	1		1						2									1	1	8
15	1		1	*2	1	1															6
16					1	1		2					1		1					1	7
17	1		1		1		2							1			1				7
18	1	1	*2													1	1				6
19	*2		1			1	1			1		1					1	1			9
20			*2	1		1	1														5
21		2			1				1		1	1		1			1				8
22	1	1	*2		*2			1					1			1	1				10
23			1		1	1						1		1							5
24			1		2		1	1			1			1					1		8
25	1	2				2	1					1						1			8
26		2											1				1				4
27	1	1				1		1		1							1				6
28	1	1	1	1	1	1	2											1			9
29	1	*2	*2	2			1										1				9
30	2		1			1															4
31	1		2	1						1				1			1				7
32	*2		1		1				1												5
33	1		1	1												1					4
34		1	1		*2	1		1								1		1			8
35	1	1	*2			1						1				1					7
36	1	1	*2																		4
37	*2																*2				4
38	1			1	1								2			1	1				7
40	1	2										1									4
41													*2	1							3
42	1					1	1			1	1					1				1	7
43	2	1	1																		4
44	1			2	1																4
45		*2	1			1															4
46	1	2	1		1								1								6
47	1	1	1	1		1				1						1					7
49			*2				1							1					1		5
50	1		*2			1		1									1				6
52		2	1														1				4
53		1	*2								1										4
54	1	1	1										*2						1		6
Total	42	36	50	19	24	21	17	9	8	11	5	7	15	7	3	10	14	5	4	5	312
pEPIs per chromosome	0.8	0.7	0.9	0.4	0.5	0.4	0.3	0.2	0.2	0.2	0.1	0.1	0.3	0.1	0.1	0.2	0.3	0.1	0.1	0.1	5.9

Table S-2 FISH analyses of metaphase spreads of pEPI transfected CHO C400 cells.
(* in the table indicates chromosomes on which two vector molecules were attached to both chromatids at identical position)
