# Supplementary material for: Establishment and mitotic stability of an extra-chromosomal mammalian replicon
Source: BMC Cell Biol. 2007 Aug 6;8:33. doi: 10.1186/1471-2121-8-33 (PMC1959191; doi:10.1186/1471-2121-8-33)
Supplement: Additional file 5 — Table S-3. Distribution of pEPI molecules to daughter nuclei after mitosis. [file 1471-2121-8-33-S5.pdf]

<u>Additional file 5</u>: **Establishment and mitotic stability of an extra-chromosomal mammalian replicon**

Isa M. Stehle, Jan Postberg, Sina Rupprecht, Thomas Cremer, Dean A. Jackson and Hans J. Lipps

Postmitotic distribution of pEPI molecules

Segregation of vector molecules to two daughter cells in which cytokinesis was not yet completed was determined by 3D-FISH analysis using pEPI as a probe. 25 pairs of cells were evaluated. We found that the number of episomes in the daughter nuclei was identical or differed by only 1 in 84% (42/50) of cases (Table S-2). In almost all cases we observed a striking mirror symmetry in the distribution of the episomes. This confirms the surprising efficiency with which the vector molecules segregate to the nuclei of daughter cells.

Postmitotic / early G1 cells	pEPI molecules per daughter nucleus		Irregular pEPIs
	nucleus A	nucleus B	
1	10	11	1
2	7	7	0
3	3	3	0
4	4	3	1
5	4	5	1
6	3	3	0
7	6	7	1
8	9	7	2
9	7	8	1
10	3	2	1
11	4	4	0
12	3	4	1
13	12	9	3
14	4	4	0
15	4	5	1
16	4	5	1
17	9	9	0
18	9	10	1
19	5	3	2
20	7	6	1
21	10	11	1
22	4	4	0
23	3	4	1
24	11	9	2
25	3	4	1
	148	147	23
pEPI Molecules per cell	5.92	5.88	0.92

Table S-3 Distribution of pEPI molecules to daughter nuclei after mitosis.
